# Oligopeptide Sortase Inhibitor Modulates *Staphylococcus aureus* Cell Adhesion and Biofilm Formation

**DOI:** 10.3390/antibiotics11121836

**Published:** 2022-12-17

**Authors:** Svetlana A. Bozhkova, Ekaterina M. Gordina, Dmitry V. Labutin, Konstantin V. Kudryavtsev

**Affiliations:** 1 Vreden National Medical Research Center of Traumatology and Orthopedics, 195427 St. Petersburg, Russia; 2 Laboratory of Molecular Pharmacology, Pirogov Russian National Research Medical University, 117997 Moscow, Russia

**Keywords:** bacterial resistance, virulence, *S. aureus*, bacterial adhesion, sortase, inhibitor

## Abstract

Prevention of bacterial adhesion is one of the most important antivirulence strategies for meeting the global challenge posed by antimicrobial resistance. We aimed to investigate the influence of a peptidic *S. aureus* sortase A inhibitor on bacterial adhesion to eukaryotic cells and biofilm formation as a potential method for reducing *S. aureus* virulence. The pentapeptide LPRDA was synthesized and characterized as a pure individual organic compound. Incubation of MSSA and MRSA strains with LPRDA induced a subsequent reduction in staphylococcal adhesion to Vero cells and biofilm formation, as visualized by microscopic and spectrophotometric methods, respectively. LPRDA did not have a cytotoxic effect on eukaryotic or bacterial cells. The pentapeptide LPRDA deserves further investigation using in vitro and in vivo models of Gram-positive bacteriemia as a potential antibacterial agent with an antiadhesive mechanism of action.

## 1. Introduction

Bacterial resistance to existing antibiotics has led to the consideration of alternative treatment options beyond the development of novel antibiotic agents. Influencing bacterial virulence factors, particularly by reducing evolutionary pressure on pathogenic bacteria and preserving the commensal microbiota, has definite advantages over traditional antibiotics [1]. *Enterococcus faecium*, *Staphylococcus aureus* (*S. aureus*), *Klebsiella pneumoniae*, *Acinetobacter baumannii*, *Pseudomonas aeruginosa*, and *Enterobacter* sp. form a group of high-priority pathogens called ESKAPE bugs (they “escape” being killed by antibiotics) that are associated with a high mortality risk and significant economic costs due to antimicrobial resistance [2]. The specific nature of different bacterial virulence mechanisms provides options for developing highly personalized antimicrobial therapy options. Adhesins, toxins, secretion systems, siderophores, immune evasion and modulation factors, and biofilm formation factors are primary targets for potential antivirulence therapies [1]. *S. aureus*, particularly in its methicillin-resistant form (MRSA), is one of the most problematic hospital pathogens. It causes respiratory tract, surgical site, prosthetic joint, and cardiovascular infections, as well as nosocomial bacteremia and severe skin infections. Resistance to other antibiotics (e.g., mupirocin, [3] which is used to eradicate *S. aureus* on mucous membranes before surgical interventions) has been observed and can be life-threatening. Systemic *S. aureus* infection is always caused by bacterial rupture through the epithelial protective layer by means of immune evasion, adhesion, and subsequent structural processes, including tissue invasion [4]. The adherent state promotes bacterial survival and triggers pathogenesis. It is obvious that preventing adhesion at an early stage following exposure of a host to a pathogen could mitigate the infection [5]. Potential strategies to reduce bacterial adhesion would apply the physical principles of blocking host or pathogen receptors with complementary ligands, inhibiting crucial biological pathways involved in biosynthesis, reducing translocation, and preventing the surface assembly of bacterial adhesin subunits [6]. Bacterial adhesins are regularly located in pili multi-subunits covalently linked to the cell wall (e.g., sortase-dependent pili) or associated with the cell membrane by non-covalent interactions [7]. Possible host cell receptors for pilus adhesins include proteins of the extracellular matrix (ECM), such as collagen, fibronectin, fibrinogen, laminin, carbohydrate moieties, and other molecules on the host cell’s surface. Non-piliated bacteria interact with host patches by means of cell wall-anchored (CWA) proteins. This is the case for *S. aureus*, which can express up to 24 different CWA proteins, among which the microbial surface component recognizing adhesive matrix molecule (MSCRAMM) family is the most prevalent [8]. The virulent nature and disease promotion of CWA proteins have been demonstrated by inactivating the corresponding bacterial gene expression and subsequently studying the infectious processes of the defective pathogens [8]. MSCRAMMs have been identified in many Gram-positive bacteria, such as different staphylococci (including coagulase-negative species), enterococci, and streptococci, and contain a universal sorting signal consisting of the pentapeptide LPXTG (Leu-Pro-X-Thr-Gly, where X indicates any amino acid) motif, a hydrophobic domain, and a charged tail at the carboxyl terminus. Host ligands induce conformational changes in MSCRAMMs, and multiple backbone–backbone hydrogen bonds stabilize bacterial adhesion via dock-lock-latch (DLL) and collagen hug (CH) mechanisms [9]. As indispensable participants in adhesion, MSCRAMMs ensure bacterial survival in the circulation during bacteremia and in the formation of abscesses in the skin and internal organs. Infective endocarditis, nasal colonization, atopic dermatitis, catheter-associated urinary tract infections, and skin abscess formation caused by *S. aureus* have been studied with different models that have revealed certain MSCRAMM-induced processes promoting pathogenesis [9].

Potential antiadhesive therapeutics can be classified into several major categories, depending on their mechanism of action [10]. The family of bacterial enzymes with cysteine protease and transferase functions that are involved in cell wall anchoring of surface proteins and pili is termed sortases [11]. *S. aureus* class A sortase (SrtA) cleaves the LPXTG motif between the threonine (T) and glycine (G) residues of MSCRAMM precursors and attaches the cleaved fragment containing the threonine residue to the pentaglycine cross-bridge of lipid II, which is a precursor in cell wall synthesis. Both strategies of genetic *srtA* knock out or inhibition of SrtA with developed inhibitors cause virulence attenuation of *S. aureus* and other Gram-positive pathogens by the defective display of surface proteins [12,13,14,15]. Several classes of SrtA inhibitors have been identified and studied using rational design and high-throughput screening (HTS) programs [12,13,14,15]. Moreover, the influence of sortases and their inhibition over the in vivo pathogenesis of pneumonia, septic endocarditis, septic arthritis, mastitis, and cutaneous and gastrointestinal infections have previously been demonstrated, with an obvious reduction in incidence rates, by suppressing the functionality of sortases [16]. SrtA inhibitors have also been shown to decrease the formation of bacterial biofilms on the host cell surface and in the ECM, since organized microbial assemblies require the surface presentation of CWA proteins [12,13].

Only a few SrtA inhibitors have been evaluated in vivo, and so far, none have advanced to clinical trials, which would necessitate an actual efficacy evaluation and follow-up of the in-depth studies of these compounds [17]. Recently, Wang et al. discussed the oligopeptidic SrtA inhibitor LPRDA, which has been studied not only in in vitro enzyme assays but has also demonstrated protection against *S. aureus*-induced mastitis in a mouse model [18]. Due to our interest in the development of novel antibacterials with antiadhesive mechanisms of action [19], we selected a reference compound with confirmed activity and further potential for development. We previously demonstrated antiadhesive activity in a dual-cell assay [19] and predicted the properties of SrtA inhibitors [20] for some compounds from our in-house collection. After analyzing the available body of data [12,13,14,15,16,17], we concluded that the oligopeptide LPRDA has certain advantages over other SrtA inhibitors, primarily owing to good solubility, in vivo activity, and being a starting point for peptidomimetic development with modified properties. We synthesized a stable, pure, individual sample of LPRDA and studied its influence on the adhesion of typical *S. aureus* strains to eukaryotic species using a dual-cell assay. To the best of our knowledge, decreased adhesion between staphylococci and eukaryotic cells caused by SrtA inhibitors has never been observed. We also showed that both prokaryotic and eukaryotic cell lines were viable under LPRDA influence and demonstrated LPRDA’s biofilm inhibitory properties. Our recent studies have revealed novel features in ligand–host interactions between LPRDA and *S. aureus* SrtA [21], which, in combination with the results of this study, could create a platform for more sophisticated antibacterial development. Most studies related to SrtA inhibitors and CWA proteins have been confined to a few laboratory strains. Research efforts must be extended to clinical strains, where considerable variation in both the repertoire of CWA proteins and sequence variation in binding domains prevail [8]. It is of note that the community-associated MRSA strain USA300 was used in preliminary LPRDA evaluation [18]. Our studies used both methicillin-susceptible and methicillin-resistant *S. aureus* ATCC strains.

## 2. Results

### 2.1. LPRDA Synthesis and Characterization

The pentapeptide LPRDA, consisting of five linked L-amino acid residues (Leu-Pro-Arg-Asp-Ala), is a partial structural analog of the LPXTG sorting signal. It was synthesized via solid-phase peptide synthesis using the 9-fluorenylmethoxycarbonyl chemistry method [22]. We carefully characterized the studied LPRDA sample using nuclear magnetic resonance (NMR) spectroscopy, high-resolution mass spectrometry (HRMC), and high-performance liquid chromatography (HPLC), and confirmed that all biological experiments were performed with an individual compound fully corresponding to the declared pentapeptide structure in the acetate form with a purity of 98+%.

### 2.2. Study of LPRDA Bactericidal Activity

**Figure 1:**
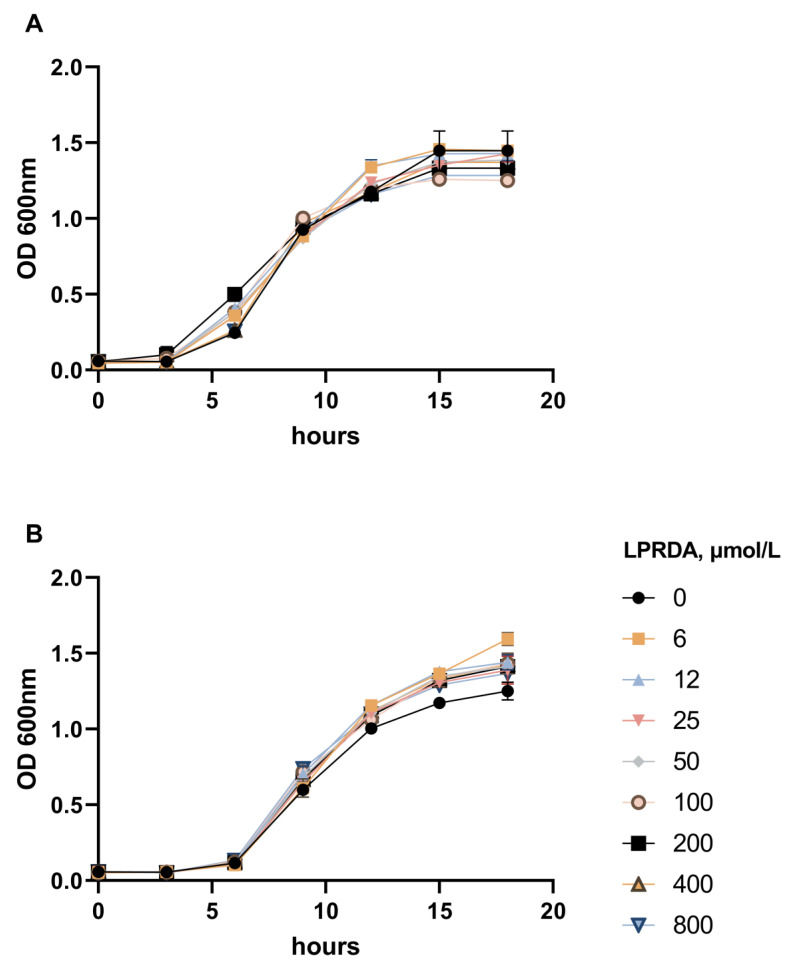
The growth curves of *S. aureus* ATCC 29213 (MSSA) (**A**) and *S. aureus* ATCC 43300 (MRSA) (**B**) biomass with various concentrations of the oligopeptide LPRDA.

Two *S. aureus* strains were used in this study: the methicillin-susceptible *S. aureus* ATCC 29213 (MSSA) strain and the methicillin-resistant *S. aureus* ATCC 43300 (MRSA) strain. Both strains were grown in the presence of LPRDA at concentrations ranging from 6 µmol/L to 800 µmol/L. We did not detect any obvious bactericidal effect of LPRDA compared to the control studies (Figure 1).

### 2.3. Adhesion of S. aureus Strains Modified by LPRDA to Vero Cells

The influence of LPRDA on staphylococcal adhesive properties was evaluated using preliminary incubation of the studied bacterial strains with LPRDA for 18 h and subsequent microscopic detection of adhesion cases of the modified staphylococci to African green monkey kidney epithelial cells (Vero) [19]. The following parameters were determined from the adhesion experiments: adhesion index (AI), defined as the average number of attached bacterial cells per eukaryotic cell; percentage of infected cells (PI), defined as the proportion of Vero cells with bacteria adhered to their surface; microbial load (ML), defined as a product AI×PI; and adhesion inhibition index (AII), defined as the ratio of experimental AI to control.

**Figure 2:**
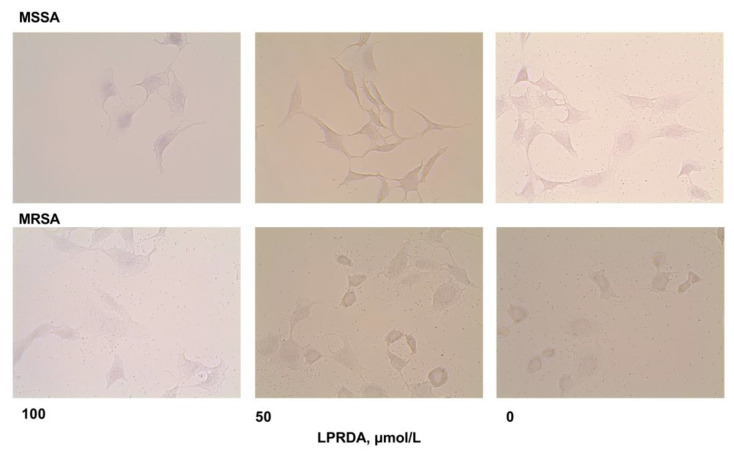
Microphotographs of *S. aureus* ATCC 29213 (MSSA) and *S. aureus* ATCC 43300 (MRSA) interactions with Vero cells (at 40-fold magnification) after preliminary incubation of staphylococci with LPRDA.

We found that the oligopeptide LPRDA had a considerable antiadhesive effect in experiments with MSSA *S. aureus* ATCC 29213 (Table 1). Incubation of these bacterial cells with 100 µmol/L of LPRDA decreased the number of eukaryotic cells affected by the bacteria; PI decreased by 16% (*p* = 0.021) relative to the control, ML decreased by 45% (*p* = 0.009), and AI decreased by 34.6% (*p* = 0.021). A two-fold decrease in the concentration of LPRDA being incubated with the bacteria significantly reduced AI (*p* = 0.01) and ML (*p* = 0.002), while the number of impaired eukaryotic cells remained essentially unaltered (but lower than the control experiment) (Table 1, Figure 2). AII was 65.4% for 100 µmol/L of LPRDA and 55.8% for 50 µmol/L of LPRDA. A further decrease in LPRDA concentration did not have a notable antiadhesive effect on the MSSA *S. aureus* ATCC 29213 strain.

Incubation of LPRDA with MRSA *S. aureus* ATCC 43300 did not induce an appreciable antiadhesive effect (Table 1, Figure 2). Under all studied LPRDA concentrations, we detected lesions in the majority of eukaryotic cells in a monolayer with staphylococci (Figure 2). The AI (*p* = 0.01) and ML (*p* = 0.01) parameters for the MRSA cell strain were approximately 10% lower than the control experiments, with reasonable significance under the highest studied concentration of LPRDA.

### 2.4. Biofilm Formation Assays

**Figure 3:**
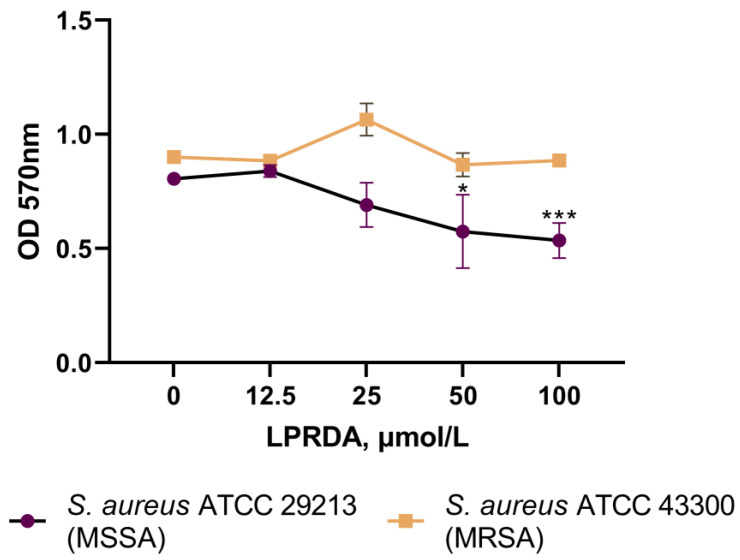
Biofilm growth of *S. aureus* ATCC 29213 (MSSA) and *S. aureus* ATCC 43300 (MRSA) strains after incubation with different LPRDA concentrations. * *p* < 0.05; *** *p* < 0.001 compared to control.

The studied *S. aureus* typical strains are classified as moderately producing biofilm strains. For the *S. aureus* ATCC 29213 (MSSA) strain, a 10-fold decrease in sessile cell bacteria number was detected after 24 h of incubation with LPRDA at 100 µmol/L concentration (Figure 3). The number of bacterial cells unaffected by LPRDA in the control wells reached 5 × 10^6^ CFU/mL, while the corresponding amount in the experimental wells incubated with LPRDA and staphylococcal cells was only 5 × 10^5^ CFU/mL. The antibiofilm effect of LPRDA on the *S. aureus* ATCC 29213 (MSSA) strain was also observed at lower LPRDA concentrations of 50 and 25 µmol/L in a concentration-dependent manner (Figure 3). No significant difference in the number of sessile bacteria was registered between the control and the *S. aureus* ATCC 43300 (MRSA) cells incubated with LPRDA (Figure 3).

**Figure 4:**
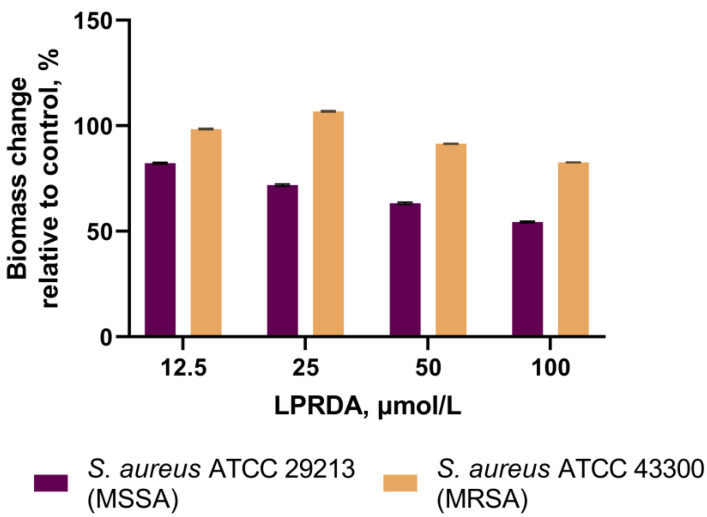
Inhibition of *S. aureus* biofilm formation relative to the control after incubation with various concentrations of the oligopeptide LPRDA.

The *S. aureus* ATCC 29213 (MSSA) strain incubated with LPRDA demonstrated a 10–44% reduction in biofilm formation compared to the untreated MSSA bacteria (Figure 4). LPRDA did not affect the biofilm formation process substantially in the case of the *S. aureus* ATCC 43300 (MRSA) strain, with a maximum decrease of 12% at 100 µmol/L LPRDA concentration (Figure 4).

### 2.5. Viability of Vero Cells in the Presence of LPRDA

**Figure 5:**
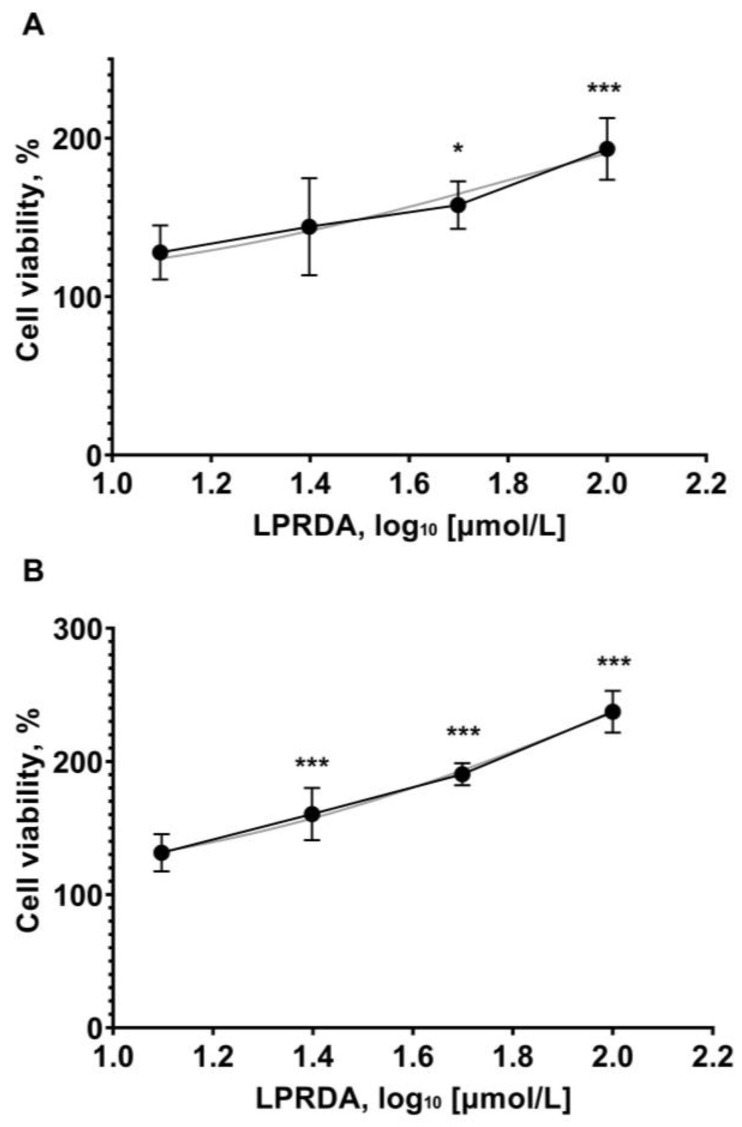
Viability of Vero cells after 72 h of incubation with LPRDA. Initial cell seeding concentration (**A**) 2000 cells/well and (**B**) 1000 cells/well. Dots represent LPRDA concentrations (12.5, 25, 50, and 100 µmol/L). * p < 0.05; *** *p* < 0.01.

In the final step, we studied whether LPRDA had any potential toxicity in relation to eukaryotic cells. Vero cells were incubated with the tested concentrations of LPRDA (12.5, 25, 50, and 100 µmol/L) for 72 h. During this period, all the tested LPRDA concentrations increased the percentage of viable Vero cells in a dose-dependent manner (Figure 5), indicating that LPRDA is nontoxic at the studied concentrations. At the same time, the trend of increasing cell viability was similar for both of the studied initial cell seeding concentrations.

## 3. Discussion

Peptide drug development has been established as a promising direction for innovative therapeutics, with 33 non-insulin peptide drugs approved worldwide since 2000, and 170 peptides in active clinical development at the moment [23]. As native modulators and ligands of pharmaceutically relevant enzymes, peptides are widely used for biochemical functional studies and the creation of enzyme inhibitors. The main limitations of peptide drug development are membrane impermeability and poor in vivo stability. Most peptides in active clinical development are aimed at extracellular targets [23].

Although the interaction between the LPXTG motif of the CWA sorting signal and the *S. aureus* SrtA active site is well known, Wang et al. designed and studied the pentapeptide LPRDA as an *S. aureus* SrtA oligopeptidic inhibitor, with a 10.61 µM of IC_50_ value inhibition of the recombinant enzyme [18]. The original report contains no characterization data for LPRDA. Moreover, the authors mentioned in the experimental section that “to improve its pharmacokinetics and therapeutic efficacy, the oligopeptide was modified by PEG2000 modification and amide modification at the N- and C-termini, respectively” [18]. There were also inconsistencies in the molecular modeling of interactions of the pentapeptide LPRDA with the *S. aureus* SrtA active site, which prompted us to perform our own in-depth theoretical analysis [21].

We recently demonstrated that incubation of the *S. aureus* ATCC 29213 (MSSA) strain with small-molecule compounds from our in-house collection attenuated subsequent bacterial adhesion to Vero cells, which was in contrast to untreated staphylococci [19]. Furthermore, we predicted the properties of SrtA inhibitors for the same compounds [20]. Establishing a correlation between the indicated research results would improve translation processes related to the development of antivirulence antimicrobials, which prompted the current study of the pentapeptide LPRDA as a reference compound with both antiadhesive and sortase-inhibiting properties. The combination of target-based and whole-cell screens often makes the discovery of new antibacterial agents more efficient [24]. Typically, researchers have been studying adhesion of bacteria to fibronectin [18], which could potentially camouflage multiple other adhesive mechanisms [9]. Several researchers have performed quantified investigations of the adhesion of *S. aureus* cells to epithelial cells using microscopic or radioactively labeled bacterial methods [25]. Recently, researchers developed a high-throughput microtiter plate-based phenotypic assay that applied fluorescence labeling of eukaryotic cell nuclei and bacteria after their adhesion and quantified the adhesion of *S. aureus* to human epithelial cells, but screening of four thousand compounds did not lead to an effective in vivo candidate [25]. We consider our semi-automatic method of detecting staphylococci adhered to eukaryotic cells to be a feasible and direct method of observing binding events. Furthermore, we recognized a target partner for a potential antiadhesive agent in intercellular interactions by preliminary incubation of bacterial cells with the testing compounds. This issue is poorly represented in the available methods [25], which may limit the establishment of potential targets.

As we demonstrated with bacterial growth assays for both a methicillin-susceptible *S. aureus* ATCC 29213 (MSSA) strain and a methicillin-resistant *S. aureus* ATCC 43300 (MRSA) strain, LPRDA did not influence bacterial viability up to 800 µmol/L concentration (Figure 1). This concentration is at least eight times higher than those used for inducing antiadhesive effects, which is consistent with the LPRDA MIC > 200 µmol/L reported in [18]. A lack of bactericidal activity is an essential property for antivirulence therapeutics [1,12,13,14,15]. The following experiments have found differences in the biological effects of LPRDA on staphylococci, depending on the strain’s nature. Wang et al. used the community-associated MRSA strain USA300 in their study and reported strong LPRDA adherence to bovine fibronectin [18]. The authors incubated the corresponding bacterial cells with LPRDA before this experiment, but the incubation times and concentrations were not reported. In our study, LPRDA-treated *S. aureus* ATCC 29213 (MSSA) cells, after a 1 h incubation period with Vero cells, showed significant attenuation of adhesive parameters, with the maximum effect at 100 µmol/L LPRDA concentration (Table 1, Figure 2). The average number of attached bacterial cells per eukaryotic cell (AI) and the proportion of Vero cells with bacteria adhering to their surface (PI) were reduced by 1.5 times and 16%, respectively, under these conditions (Table 1). LPRDA at a concentration of 50 µmol/L used for incubation induced virtually the same effect, but the PI data were insignificant. Based on the results we obtained, we can hypothesize that the pure pentapeptide LPRDA caused defective representation of MSCRAMMs on the bacterial surface, which appeared as a decrease in heterogenic cell-binding events under microscopic visualization. Additionally, as is evident from our experimental data, LPRDA induced a much less noticeable effect on MRSA *S. aureus* ATCC 43300 cells (Table 1, Figure 2). It is clear that the repertoire of CWA proteins [8,9] varies considerably among *S. aureus* bacterial strains, and characterizing definite species in certain strains remains a challenging task.

Incubation of LPRDA with MRSA *S. aureus* ATCC 43300 cells did not induce a significant antiadhesive effect (Table 1, Figure 2). At all studied LPRDA concentrations, we observed that the majority of eukaryotic cells in a monolayer were affected with staphylococci (Figure 2). The AI (*p* = 0.01) and ML (*p* = 0.01) parameters for the MRSA cell strain were lower than those in the control experiments by approximately 10%, with reasonable significance under the maximum studied LPRDA concentration.

CWA proteins in Gram-positive bacteria are involved in multiple functions, although some are redundant [8]. The MSCRAMM serine-aspartate repeat-containing protein C (SdrC) and the fibronectin-binding proteins (FnBPs) are involved in homophilic interactions that promote the aggregation of cells and contribute to biofilm formation [9]. Multiple interactions between MSCRAMMs on adjacent cells cumulatively result in strong cell–cell binding. The prevention of biofilm formation is recognized as a desired antivirulence strategy to limit the progression of chronic bacterial diseases [17]. *S. aureus* biofilms contribute to the severity and progression of prosthetic joint infections, which requires the development of novel therapeutic approaches [26,27,28]. *S. aureus* clinical isolates generate at least two distinct types of biofilm mediated by the FnBPs or the *icaADBC*-encoded polysaccharide intercellular adhesin (PIA) [29]. MRSA biofilms are FnBP-dependent and *ica* independent, while PIA production stimulates MSSA biofilm development [29]. Moreover, the LPXTG-containing CWAs Bap, Aap/SasG, SasC, and protein A are known mediators of biofilm development. Protein A122 was established as the most abundant CWA protein in the community-associated MRSA strain LAC, whereas hospital-associated MRSA strains expressed high levels of FnBPs [8]. The combination of biofilm-destroying compounds and conventional antibiotics is a prospective strategy to combat biofilm-associated infections [30].

Wang et al. studied the community-associated MRSA strain USA300 and its *srtA* mutants in a biofilm formation assay [18]. They established that LPRDA at a concentration of 25 µmol/L attenuated biofilm formation to the level of knocked out bacteria [18]. We studied the influence of LPRDA at concentrations of 12.5 to 100 µmol/L on biofilm production by the MSSA and MRSA strains used in this adhesion experiment. We observed a concentration-dependent decrease in the amount of biofilm formed by the *S. aureus* ATCC 29213 (MSSA) strain when it was incubated with increasing concentrations of LPRDA (Figure 3 and Figure 4). The result for the *S. aureus* ATCC 29213 (MSSA) strain biofilm is similar to the one reported for MRSA strain USA300 [18]; a 44% reduction in biofilm was achieved when it was incubated with LPRDA at a concentration of 100 µmol/L (Figure 4). In the case of the *S. aureus* ATCC 43300 (MRSA) strain, the biofilm formation process was affected substantially less, with a 12% decrease in biomass at 100 µmol/L LPRDA concentration (Figure 4).

Considering the available data on CWA protein biofilm formation functions [8,9,26,27,28,29], we can conclude that the pentapeptide LPRDA disrupted the biofilm formation process of both the MSSA and MRSA strains by affecting the expression of the corresponding MSCRAMMs on the bacterial cell walls. Moreover, *S. aureus* adhesion to eukaryotic cells correlated positively with biofilm-forming ability: the decrease in heterophilic cell interactions caused by elevated LPRDA concentrations matched the decrease in biofilm formation induced by homophilic bacterial cell interactions. From our experiments, we also observed that the MSSA strain was more susceptible to the influence of LPRDA than the MRSA strain.

As direct toxicity data were unavailable for LPRDA, we undertook appropriate experiments to fill this gap. Vero cells were seeded at concentrations of 1000 and 2000 cells/well and incubated with the working concentrations of LPRDA up to 100 µmol/L for 72 h. Eukaryotic cell viability increased in an LPRDA dose-dependent manner in all the studied cases (Figure 5).

## 4. Materials and Methods

### 4.1. Reagents, Cell Cultures, and Strains

The pentapeptide LPRDA was synthesized using the solid-phase peptide 9-fluorenylmethoxycarbonyl chemistry method [22]. Colorless light solid, soluble in water: 98.4% de (HPLC, Phenomenex Kinetex EVO C18, *t_R_* 8.18 min). HRMS (ESI, *m*/*z*): [*M* + H]^+^ calculated for C_24_H_42_N_8_O_8_, 571.3204; found, 571.3188.

The Vero cell line (African green monkey kidney epithelial cells) was obtained from the Common Use Center Vertebrate Cell Culture Collection, Institute of Cytology, Russian Academy of Sciences (Moscow). Vero cells were grown in standard Dulbecco’s Modified Eagle’s Medium (DMEM) with 4.5 g/L glucose, L-glutamine, sodium pyruvate, 10% fetal calf serum, penicillin 100 U/mL, and streptomycin 100 µg/mL (Capricorn Scientific GmbH, Ebsdorfergrund, Germany). The medium was replaced every 3 days, and cells were split 1:3.

The two *Staphylococcus aureus* strains used in the study were methicillin-susceptible *Staphylococcus aureus* ATCC 29213 (MSSA) and methicillin-resistant *Staphylococcus aureus* ATCC 43300 (MRSA).

### 4.2. S. aureus Viability in the Presence of LPRDA

The bactericidal activity of LPRDA was determined according to the Clinical and Laboratory Standards Institute (CLSI). Inoculums of the tested strains were dispersed in Mueller–Hinton broth (MHB) to obtain a suspension of 1 × 10^5^ CFU/mL, which was distributed in 96-well plates with 150 μL of MHB and LPRDA concentrations of 6–800 µmol/L. Vancomycin was used as a reference antibacterial drug. Raw culture medium was included to check the sterility and used as a negative control. The plates were incubated at 37 °C for 18 h (20 cycles, 3600 s, OD_600_) in a SPECTROstar NANO spectrophotometer (BMG Labtech, Ortenberg, Germany). The curves were analyzed using SPECTROstar NANO MARS software v5.5.

### 4.3. Bacterial Adhesion to Vero Cells

Vero cells were seeded in 96-well plates at 25 × 10^3^ cells/well and grown overnight. Bacterial cells (*S. aureus* ATCC 29213 or *S. aureus* ATCC 43300) were incubated with the oligopeptide LPRDA in the concentration range 12.5–100 µmol/L at 37 °C for 18 h with constant shaking at 100 rpm. Luria–Bertani (LB) medium and untreated bacteria served as a control. After incubation, the bacterial suspension was centrifuged at 10,000× *g* rpm for 10 min and washed twice with sterile PBS for 5 min at 10,000 rpm. The final bacterial concentrations were adjusted to the OD_560_ of 0.3 McFarland in sterile saline. Next, the monolayer of Vero cells in the 96-well plates was inoculated with 100 μL of the bacterial suspension with an equal volume of the stock DMEM culture medium and incubated for 1 h. The monolayer was washed three times with phosphate-buffered saline (PBS), followed by fixation with 4% formaldehyde for 60 min. Then, the wells of the plates were washed twice with PBS, dried, and stained with purified gentian violet solution (1%) for 15 min. The dye was removed, and images were captured in 20 random fields at 40× using an Evos FL microscope (Thermo Fisher Scientific, Waltham, MA, USA). Images were captured in 4–10 random fields of view at 40-fold magnification using an Evos FL microscope. Images were analyzed using ImageJ Fiji software v1.53c. 

### 4.4. Inhibition of Biofilm Formation Assay

The assay was performed according to the protocol described by O’Toole, with minor modifications [31]. To evaluate the effect of LPRDA on biofilm formation by the *S. aureus* ATCC 29213 and *S. aureus* ATCC 43300 strains, 150 µL of sterile LB broth with 4% glucose containing 12.5–100 µmol/L concentrations of LPRDA was added to four wells of 96-well plates. Then, 50 µL of staphylococcal culture (1 × 10^7^ CFU/mL) was added and incubated at 37 °C for 24 h. LB culture broth that was not treated with LPRDA was used as the positive control. After 24 h, the plates were washed with PBS to remove planktonic and dead cells, stained with a 0.1% solution of gentian violet for 20 min, followed by extraction of the bound dye with 96% ethanol. The optical density (OD) of the obtained extracts was measured at 570 nm using a SPECTROstar Nano spectrophotometer.

The bacterial strains were classified into categories according to the Stepanović criteria [32]. Quantitative calculation of *S. aureus* CFU in the biofilm formed in the presence of LPRDA and in the control experiments was assessed by inoculation on Columbia agar according to the Koch procedure [33]. Inhibition of biofilm formation was determined according to the Kumari method [34].

### 4.5. Eukaryotic Cell Viability Assay

Vero cells were seeded in 96-well plates at initial concentrations of 1000 and 2000 cells/well and incubated for 24 h. LPRDA was added to the wells as the DMEM solution at concentrations of 100, 50, 25, and 12.5 µmol/L. For cell growth curves, two-fold dilutions were seeded (16,000, 8000, 4000, and 2000 cells/well) and grown for 24 h in parallel.

In 72 h, 3-(4,5-dimethylthiazol-2-yl)-2,5-diphenyl tetrazolium bromide (MTT) was added to the corresponding wells at a final concentration of 1 mg/mL and incubated for 3 h. The formed formazan was dissolved in 100 µL DMSO per well for 5 min at 200 rpm in a shaker. The OD was measured at 570 and 640 nm using a SPECTROstar Nano. The OD was corrected by the differences in blank well absorption at 570–640 nm. The same experimental procedure was carried out in 24 h for growth curve construction. 

The number of viable cells was interpolated from the cell growth curves. The data were normalized as the percentage of viable cells to the control. 

### 4.6. Statistical Analysis

All experiments were performed in 3–5 independent replicates. The results were analyzed using the GraphPad Prism 9.0 program (USA). The results were calculated as means with standard deviations. The normality of the distribution was assessed using the quantitative Shapiro–Wilk method (W-test). The statistical significance of differences was assessed using Student’s *t*-test.

The adhesion parameters of *S. aureus* to Vero cells and the viability of Vero cells in wells with LPRDA and in control wells were compared using one-way ANOVA dispersion analysis with post-hoc Dunnett’s tests. Values with *p* < 0.05 were considered statistically significant.

## 5. Conclusions

To summarize, the pentapeptide Leu-Pro-Arg-Asp-Ala (LPRDA) is one of the most studied *S. aureus* sortase A inhibitors. It attenuates the virulence of both MSSA and MRSA strains in vitro and in vivo. As with other sortase A inhibitors tested in murine models of *S. aureus* infections [35,36,37,38,39,40,41], LPRDA may impact the development of novel antibacterial agents. The pure crystalline form of LPRDA is characterized by good solubility and tolerance to eukaryotic and bacterial cells at working concentrations. In addition to the previously reported reduction in binding to fibronectin and in vivo efficacy on a mastitis model for the modified oligopeptide, we demonstrated attenuation of heterophilic and homophilic bacterial cell interactions obviously caused by defective CWA protein exposition induced by the pure pentapeptide LPRDA. According to our experiments, the MSSA strain is more susceptible to LPRDA influence than the MRSA strain. *S. aureus* adhesion to Vero cells and biofilm formation were reduced in a dose-dependent manner after the bacterial cells were incubated with LPRDA.

## Figures and Tables

**Table 1:** Adhesion parameters of *S. aureus* strains modified by LPRDA to Vero cells.

*S. aureus* Cell Strain	LPRDA Concentration (µmol/L)	AI (Mean ± SD); *p* *	PI (Mean ± SD,%); *p* *	ML (Mean ± SD); *p* *
ATCC 29213 (MSSA)	100	3.4 ± 0.4; 0.021	84.0 ± 2.8; 0.021	286.9 ± 38.8; 0.009
	50	2.9 ± 0.2; 0.01	85.0 ± 4.6; 0.054	240.8 ± 9.2; 0.002
	25	4.8 ± 0.2; 0.43	99.0 ± 0.8; 0.373	471.1 ± 26.5; 0.414
	12.5	5.4; 0.53	100; 0.53	542.9; 0.53
	0 (control)	5.2 ± 0.3; –	100; –	516.7 ± 31.1; –
ATCC 43300 (MRSA)	100	6.8 ± 0.1; 0.01	100; –	679.2 ± 13.6; 0.01
	50	7.3 ± 0.2; 0.332	100; –	727.4 ± 18.2; 0.33
	25	7.4 ± 0.1; 0.516	100; –	744.2 ± 8.3; 0.516
	12.5	7.9; 0.056	100; –	790.2; 0.056
	0 (control)	7.6 ± 0.1; –	100; –	755.9 ± 10.6; –

* significance criteria *p* < 0.05. Abbreviations: AI—adhesion index; PI—percentage of infected cells; ML—microbial load.

## Data Availability

Not applicable.
